# Residence of the Nucleotide Sugar Transporter Family Members SLC35F1 and SLC35F6 in the Endosomal/Lysosomal Pathway

**DOI:** 10.3390/ijms25126718

**Published:** 2024-06-18

**Authors:** François Van den Bossche, Virginie Tevel, Florentine Gilis, Jean-François Gaussin, Marielle Boonen, Michel Jadot

**Affiliations:** 1 Physiological Chemistry Laboratory, URPhyM, NARILIS, University of Namur, 5000 Namur, Belgium; francois.vandenbossche@unamur.be (F.V.d.B.); virginie.tevel@unamur.be (V.T.); florentine.gilis@unamur.be (F.G.); 2 Laboratory of Intracellular Trafficking Biology, URPhyM, NARILIS, University of Namur, 5000 Namur, Belgium; jean-francois.gaussin@unamur.be

**Keywords:** lysosome, recycling endosomes, nucleotide sugar transporter, SLC35F1, SLC35F6, subcellular localization, tyrosine and dileucine sorting motifs

## Abstract

The SLC35 (Solute Carrier 35) family members acting as nucleotide sugar transporters are typically localized in the endoplasmic reticulum or Golgi apparatus. It is, therefore, intriguing that some reports document the presence of orphan transporters SLC35F1 and SLC35F6 within the endosomal and lysosomal system. Here, we compared the subcellular distribution of these proteins and found that they are concentrated in separate compartments; i.e., recycling endosomes for SLC35F1 and lysosomes for SLC35F6. Swapping the C-terminal tail of these proteins resulted in a switch of localization, with SLC35F1 being trafficked to lysosomes while SLC35F6 remained in endosomes. This suggested the presence of specific sorting signals in these C-terminal regions. Using site-directed mutagenesis, fluorescence microscopy, and cell surface biotinylation assays, we found that the EQERLL^360^ signal located in the cytoplasmic tail of human SLC35F6 is involved in its lysosomal sorting (as previously shown for this conserved sequence in mouse SLC35F6), and that SLC35F1 localization in the recycling pathway depends on two YXXΦ-type signals: a Y^367^KQF sequence facilitates its internalization from the plasma membrane, while a Y^392^TSL motif prevents its transport to lysosomes, likely by promoting SLC35F1 recycling to the cell surface. Taken together, these results support that some SLC35 members may function at different levels of the endosomal and lysosomal system.

## 1. Introduction

The vast majority of the SLC35 (solute carrier 35) family of nucleotide sugar transporters, including members of subfamilies A, B, C, and D, are involved in the translocation of nucleotide sugars across the membrane of the endoplasmic reticulum (ER) or the Golgi apparatus, in exchange for the corresponding monophosphate nucleosides [1,2,3]. The activated sugars subsequently serve as monosaccharide donors in glycan anabolism, including glycosaminoglycan synthesis, as well as N- and O-glycosylation [4,5].

Most members of the SLC35E and F subfamilies remain orphan transporters. Interestingly, Farenholtz et al. documented the presence of SLC35F1, which is mostly expressed in the brain, in recycling endosomes of neuronal cells [6], whereas our group discovered that the ubiquitously expressed SLC35F6 protein (previously referred to as P40) is localized to the membranes of lysosomes [7]. These discoveries are rather surprising, considering that glycosylation processes tend to occur in biosynthetic compartments. To date, SLC35D3 and SLC35G2 are the only other SLC35 family members reported to be present in post-Golgi vesicular structures. Qian et al. found these proteins, as well as SLC35F1, in synaptic vesicles [8]. They also discovered that SLC35D3 transports uridine diphosphate (UDP)-glucose into these vesicles, from which it may be exocytosed [8]. It was postulated that extracellular UDP-glucose could act as an extracellular signaling molecule. Of note, SLC35D3 has also been detected in early endosomes of megakaryocytes, as well as in dense granules of platelets [9,10]. It was found that activated platelets can supply UDP-sugars for extracellular glycosylation processes [11].

An open question is how several SLC35 proteins are sorted into specific post-Golgi compartments. For example, human SLC35F1 and SLC35F6 share 17.51% identity, 34.33% similarity, and comprise up to 10 putative transmembrane domains. However, the former appears to be retained in endosomes while the latter reaches lysosomes [6,7].

Newly synthesized membrane proteins can either travel directly from the trans-Golgi network (TGN) to endosomal compartments or they can follow an indirect route and be sorted at the cell surface before reaching organelles of the endocytic pathway [12,13]. From endosomes, the proteins can either reach lysosomes or be recycled back to the TGN and/or plasma membrane (PM), which limits their presence in lysosomes. The initial sorting step to endosomes is usually mediated by amino acid motifs located in a cytoplasmic region of the protein, which act as sorting signals. Indeed, they are recognized by cytosolic adaptor proteins that recruit vesicular coat components such as clathrin, leading to the packaging of the protein into transport vesicles followed by its delivery to endosomes [14]. These signals often consist of short amino acid sequences of the YXXΦ or [D/E]XXXL[L/I]-type [12]. The tyrosine-based motifs (YXXΦ, where Φ represents a bulky hydrophobic amino acid) can, for example, be recognized by AP-1, an adaptor complex active in the TGN, and/or by AP-2, which is involved in endocytosis from the plasma membrane [12,15]. The dileucine motifs ([D/E]XXXL[L/I]) can be recognized by the same adaptor complexes [15,16].

Membrane proteins that do not travel to the lysosomes after reaching endosomes (at least not for a while) are usually recognized by a recycling machinery. For instance, the retromer complex, composed of three core VPS29, VPS26A/B, and VPS35 subunits, plays a crucial role in recycling specific cargo proteins (such as the mannose 6-phosphate receptors) from endosomes to the TGN [17]. Depending on the other proteins that are part of the retromer complexes (such as sorting nexins), these complexes may also facilitate cargo recycling from endosomes toward the plasma membrane [18]. Of note, aside from their role in protein sorting toward endosomes, YXXΦ or [D/E]XXXL[L/I] signals can also act as recycling signals to the plasma membrane for some transmembrane proteins such as the Coxsackie and Adenovirus Receptor (CAR) or the MHC-I-associated invariant chain p33, respectively [19,20,21].

Interestingly, SLC35F1 and SLC35F6 contain, within their cytosolic C-terminal tail, several consensus tyrosine-(of the YXXΦ-type) and/or dileucine-based motifs. It has previously been shown that the EQERLL^360^ motif (i.e., dileucine) motif found in the C-terminal tail of mouse SLC35F6 intervenes in its targeting to lysosomes, as substitution of the leucine pair with other amino acids (L360A-L361V) resulted in the mistargeting of the protein to the plasma membrane [22]. To the best of our knowledge, the signal(s) that control(s) the subcellular trafficking of SLC35F1 has(have) not been elucidated, nor has the point of divergence in the trafficking routes of SLC35F1 and SLC35F6 been identified. Of note, this point of divergence is suggested based on the previous report of SLC35F1 presence in recycling endosomes and SLC35F6 localization to lysosomes, but it should be noted that the localization and trafficking of these proteins have not yet been studied in a common cellular model.

The present study focuses on these unresolved questions. We first compared the subcellular residence sites of human SLC35F1 and SLC35F6 (overexpressed in HeLa cells or expressed endogenously in a neuronal cell line). We then constructed chimeric SLC35F1 and SLC35F6 proteins by exchanging their C-terminal tails. We also mutated the sequences coding for putative tyrosine- and dileucine-based sorting signals. The localization and subcellular trafficking of these proteins was then assessed using a combination of fluorescence confocal microscopy and cell surface biotinylation assays. Based on these analyses, we can now report that the EQERLL signal located in the cytoplasmic tail of human SLC35F6 plays a lysosomal-sorting role. By contrast, SLC35F1 contains two tyrosine-based signals in its C-terminal tail that ensure its localization in recycling endosomes and at the cell surface by allowing the protein to escape transport to lysosomes.

## 2. Results

### 2.1. Human SLC35F1 (hSLC35F1) and SLC35F6 (hSLC35F6) Have Different Main Subcellular Localizations in HeLa Cells

**Figure 1:**
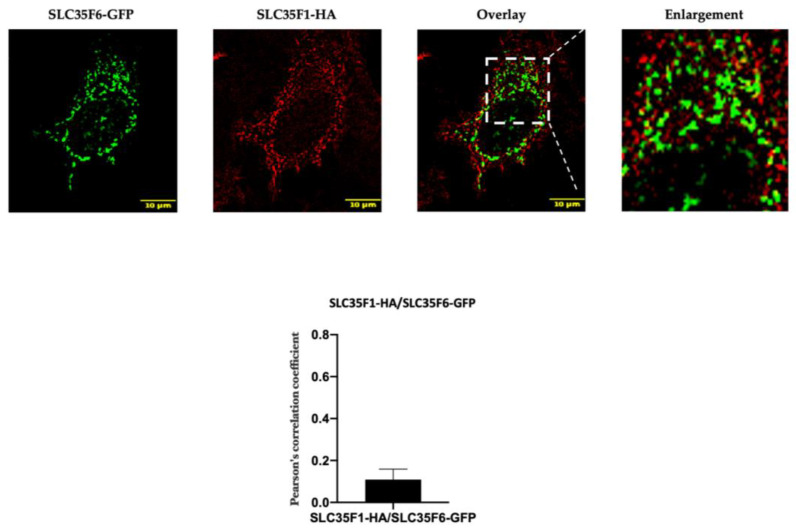
Comparison of the subcellular localization of SLC35F1-HA and SLC35F6-GFP in transfected HeLa cells. At 48 h post-transfection, cells were fixed with 4% paraformaldehyde and processed for the immunodetection of SLC35F1-HA using an anti-HA antibody (red). SLC35F6-GFP was visualized in green. Micrographs were obtained using an SP5 confocal microscope. The area delimited by the white dotted lines has been enlarged 2.5 times. The graph shows the quantification of the extent of co-localization between red and green signals using Pearson’s correlation coefficient analysis (n ≥ 10 cells quantified in each of three independent experiments).

We co-expressed a C-terminally fused HA-tagged hSLC35F1 construct and a C-terminally fused GFP-tagged hSLC35F6 construct (SLC35F1-HA and SLC35F6-GFP, respectively) in HeLa cells. At 48 h post-transfection, little co-localization was detected between these constructs, as confirmed by a low Pearson’s correlation coefficient between corresponding signals (Figure 1). In fact, while a punctate pattern was observed for both proteins, SLC35F6 signals were more concentrated in the perinuclear region compared with SLC35F1 signals, which were predominant in the cell periphery (near and possibly at the plasma membrane to some extent).

**Figure 2:**
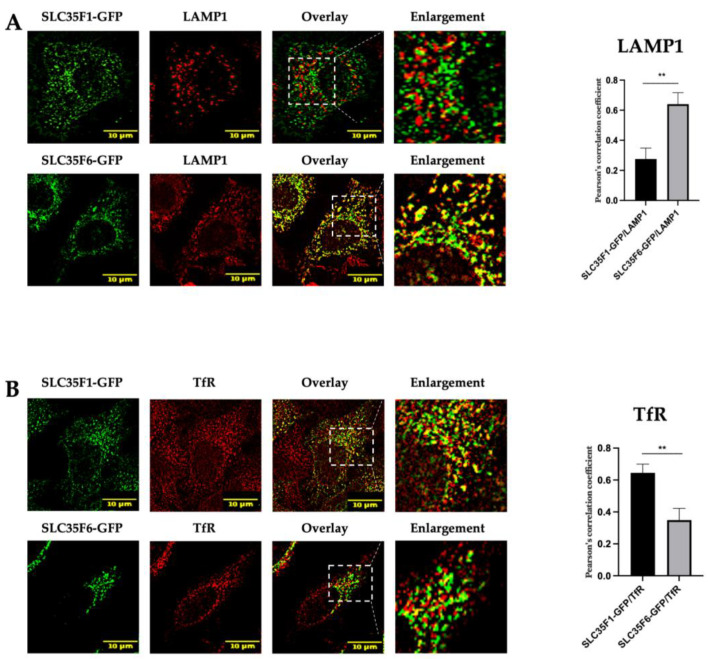
Subcellular localization of SLC35F1-GFP and SLC35F6-GFP in transfected HeLa cells. After 48 h of transfection with the GFP constructs, cells were fixed with 4% paraformaldehyde and processed for the detection of (**A**) the late endosomal/lysosomal marker LAMP1 (red) or (**B**) the recycling endosome marker TfR (red). Micrographs were obtained using an SP5 confocal microscope. Graphs show quantifications of the extent of co-localization between the protein of interest (SLC35F1 or F6) and a given marker using Pearson’s correlation coefficient analyses (n ≥ 10 cells quantified in each of three independent experiments). Unpaired *t*-test, ** *p* ≤ 0.01. The area delimited by the white dotted lines has been enlarged ~2.5 times.

Next, we conducted co-localization assays between GFP-tagged SLC35F1 or SLC35F6 constructs with the late endosomal/lysosomal marker LAMP1 or a marker for recycling endosomes, TfR (Transferrin Receptor) [23,24]. The results are presented in Figure 2. We detected an extensive co-localization between SLC35F6-GFP and LAMP1 (mean Pearson’s correlation coefficient of 0.6402) and a weaker overlap of this protein with TfR signals (0.3487), consistent with the predominant lysosomal localization that we reported previously for the mouse SLC35F6 protein [7]. By contrast, SLC35F1-GFP only slightly co-localized with LAMP1 (mean Pearson’s correlation coefficient of 0.2758) and showed a strong co-localization with TfR (mean Pearson’s correlation coefficient of 0.6448).

These results support that the main localization sites of SLC35F1 and SLC35F6 are different, the latter being enriched in late endosomes/lysosomes, whereas the former appears concentrated in TfR-positive compartments, most likely recycling endosomes. These data are consistent with the report of SLC35F1 colocalization with the recycling endosome-associated Rab11 protein in the human U251MG glioblastoma cell line [6].

### 2.2. In SH-SY5Y Neuronal Cells, Endogenously Expressed SLC35F1 and SLC35F6 Are Located in Recycling Endosomes and Lysosomes, Respectively

**Figure 3:**
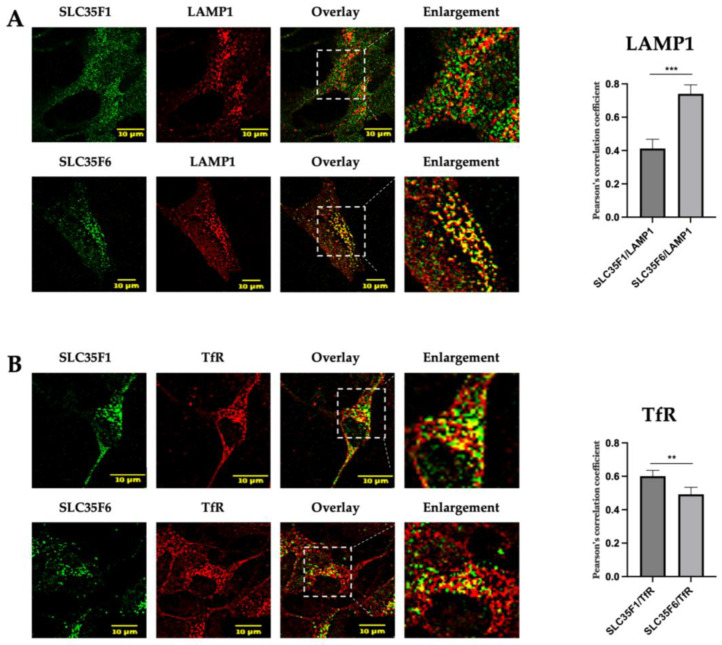
Endogenous subcellular localization of SLC35F1 and SLC35F6 in SH-SY5Y cells. Cells were fixed with 4% paraformaldehyde and processed for the immunodetection of endogenous SLC35F1 or SLC35F6 (green) and (**A**) the lysosomal marker LAMP1 (red) or (**B**) the recycling endosome marker TfR (red). Micrographs were obtained using an SP5 confocal microscope. Graphs show the quantification of co-localization extents using Pearson’s correlation coefficient (n ≥ 10 cells quantified in each of three independent experiments). Unpaired *t*-test, ** *p* ≤ 0.01, *** *p* ≤ 0.001. The area delimited by the white dotted lines has been enlarged ~2.5 times.

According to the “Human Protein Atlas”, SLC35F1 is mostly expressed in the brain, whereas SLC35F6 is ubiquitously expressed [25]. Taking this information into account, we decided to analyze the endogenous subcellular localization of these two proteins in the neuroblastoma SH-SY5Y cell line (Figure 3).

We detected a strong co-localization between endogenous SLC35F6 and LAMP1 (mean Pearson’s correlation coefficient of 0.7407), while SLC35F1 showed weaker co-localization with this marker (mean Pearson’s correlation coefficient of 0.4122) (*p* < 0.001) (Figure 3A). Conversely (Figure 3B) endogenously expressed SLC35F1 presented extensive co-localization with TfR (mean Pearson’s correlation coefficient of 0.6011), while SLC35F6 col-localization with this maker was found to be lower (mean Pearson’s correlation coefficient of 0.4926) (*p* < 0.01).

Although we could not directly assess the level of colocalization between SLC35F1 and F6 owing to the same antibody species used to detect them in neuronal cells, these results support a different primary localization for these endogenously expressed proteins, as observed after their overexpression in HeLa cells. Thus, these data indicate that tagging of these proteins with GFP (or HA) at their C-terminal end does not disrupt their subcellular trafficking to recycling endosomes and lysosomes, respectively.

### 2.3. Overexpression of Rab11 Disrupts the Intracellular Distribution of SLC35F1 but Has Little Effect on SLC35F6 Localization

Rab11 is a small GTPase involved in the recycling of various proteins from endosomes toward the plasma membrane or TGN [26]. It is notably involved in the recycling of the TfR to the plasma membrane [27,28]. Indeed, upon overexpression of wild-type (WT) Rab11, it has been reported that TfR accumulates in enlarged endosomal structures and no longer recycles to the plasma membrane, possibly owing to the fusion of sorting endosomes with recycling endosomes, which appears to disturb membrane dynamics in the TfR recycling pathway [29,30]. Since SLC35F1 largely co-localized with TfR and could follow a similar recycling route, we tested whether overexpression of a Rab11 construct (DsRed-Rab11-WT) would alter SLC35F1 distribution in the cells.

**Figure 4:**
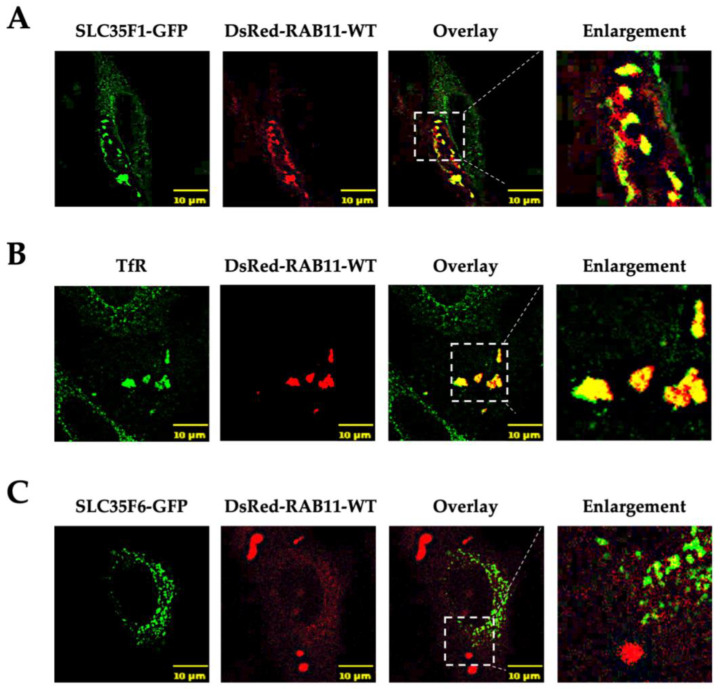
Analysis of the subcellular distribution pattern of SLC35F1-GFP and SLC35F6-GFP in HeLa cells that overexpress Rab11. At 48 h after co-transfection of DsRed-Rab11-WT with either SLC35F1-GFP (**A**) or SLC35F6-GFP (**C**), cells were fixed with 4% paraformaldehyde and observed using an SP5 confocal microscope. (**B**) Cells transfected with DsRed-Rab11-WT alone were processed for the detection of endogenous TfR (green) as a positive control. The area delimited by the white dotted lines has been enlarged ~2.5 times.

Interestingly, overexpression of the WT form of DsRed-Rab11 resulted in the appearance of very large Rab11-positive vesicles in which SLC35F1-GFP and TfR were massively accumulated (Figure 4A,B). In contrast, the distribution of SLC35F6-GFP remained mostly unchanged in Rab11 overexpressing cells (Figure 4C).

These results suggest that SLC35F1 is a cargo of a recycling pathway involving Rab11-positive recycling endosomes, while SLC35F6 is sorted into lysosomes.

### 2.4. The C-Terminal Tails of SLC35F1 and SLC35F6 Contain the Molecular Information Responsible for Their Differential Trafficking

**Figure 5:**
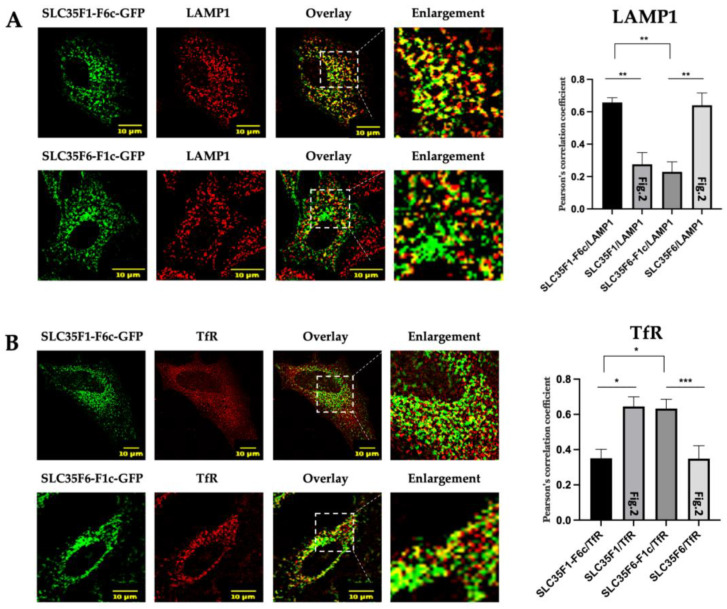
Subcellular localization of SLC35F1-F6c-GFP and SLC35F6-F1c-GFP in transfected HeLa cells. After 48 h of transfection with either SLC35F1-F6c-GFP or SLC35F6-F1c-GFP (green), the cells were fixed with 4% paraformaldehyde and processed for the detection and lysosomal marker LAMP1 (red) (**A**) or the recycling endosome marker TfR (red) (**B**). Micrographs were obtained using an SP5 confocal microscope. Graphs show the quantification of co-localization extents using Pearson’s correlation coefficient (n ≥ 10 cells quantified in each of three independent experiments). Unpaired *t*-tests, * *p* ≤ 0.05, ** *p* ≤ 0.01, *** *p* ≤ 0.001. For comparison, the colocalization analyses between the wild-type SLC35F1 and F6 proteins and the LAMP1 and TfR markers shown in Figure 2 are replotted in this figure (specified as Figure 2 inside corresponding columns). The area delimited by the white dotted lines has been enlarged ~3 times.

As the intracellular trafficking of endosomal and lysosomal transmembrane proteins is often mediated by sorting motifs located in their C-terminal cytoplasmic region, we postulated that different sorting signals could be contained in the tails of SLC35F1 and F6. To test this hypothesis, we exchanged their respective C-terminal tails (referred to as F1c or F6c, respectively) and analyzed the subcellular localization of the chimeric proteins using confocal microscopy (Figure 5).

While, as reported above, wild-type SLC35F1-GFP colocalized to a greater extent with TfR than with LAMP1 (Figure 2), we observed the opposite for the SLC35F1-F6c-GFP chimera (Figure 5A,B; for comparison, graphs in Figure 5 include the colocalization levels measured for wild-type SLC35F1, which are shown in Figure 2). These data suggest that the C-terminal region of SLC35F6 allows transport of the SLC35F1-F6c chimeric protein to lysosomes. Similarly, we found that replacement of the SLC35F6 C-terminal tail with the SLC35F1 tail (F1c) resulted in retention of the SLC35F6-F1c-GFP chimeric protein in TfR-positive recycling endosomes (Figure 5A,B).

Thus, the C-terminal tails of the two proteins carry critical information that determines their subcellular residence.

### 2.5. Two YXXΦ Sorting Motifs in SLC35F1 and a [D/E]XXXL[L/I] Motif in SLC35F6 C-Terminal Tails Are Critical for Their Respective Trafficking

The SLC35F1 C-terminal tail contains two putative YXXΦ motifs: Y^367^KQF and Y^392^TSL. The SLC35F6 tail comprises a Y^333^NGL motif and a dileucine motif, EQERLL^360^. For the mouse SLC35F6 ortholog, our team previously identified that substitution of the corresponding leucine residues, L^360^L^361^, into A^360^V^361^ leads to the accumulation of the protein at the plasma membrane, whereas mutation of the tyrosine motif (alone or in combination with the dileucine motif) has no effect [22].

To study the putative role of the tyrosine motifs located in SLC35F1 and to test if the dileucine motif of human SLC35F6 also controls its transport, we designed the following constructs: SLC35F1 with a Y^367^ substitution for alanine (SLC35F1-Y367A-GFP), SLC35F1 with a Y^392^ substitution for alanine (SLC35F1-Y392A-GFP), and an SLC35F6 construct containing L^359^L^360^ substituted by an alanine and a valine (SLC35F6-L359A-L360V-GFP). While it has been reported that substitution of a single critical leucine is sufficient to disrupt this type of dileucine signal in some proteins [12], we chose to mutate both leucine residues to ensure complete inactivation of this putative trafficking motif in human SLC35F6.

**Figure 6:**
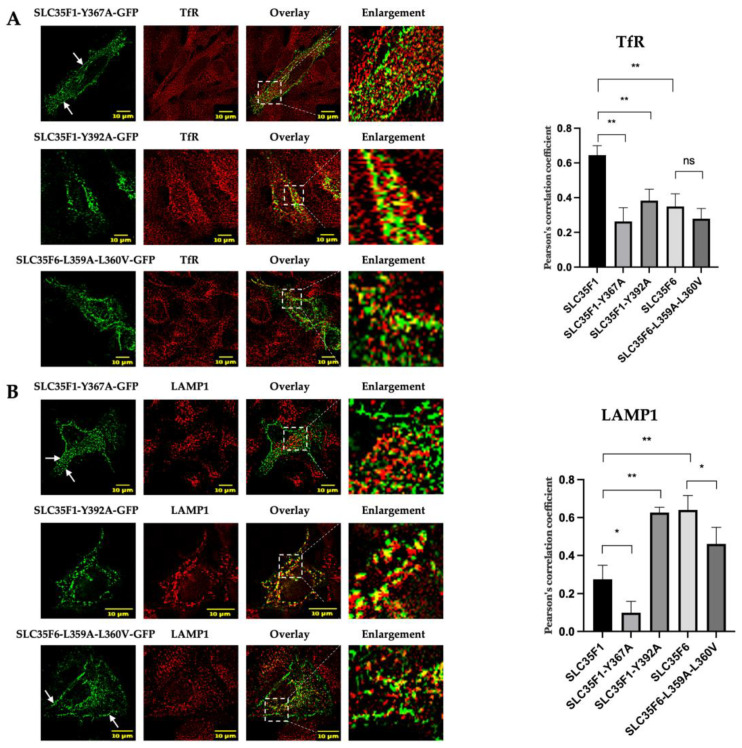
Subcellular localization of SLC35F1-Y367A-GFP, SLC35F1-Y392A-GFP, and SLC35F6-L359A-L360V-GFP in transfected HeLa cells. At 48 h post-transfection with either SLC35F1-Y367A-GFP, SLC35F1-Y392A-GFP, or SLC35F6-L359A-L360V-GFP (green), the cells were fixed with 4% paraformaldehyde and processed for the detection of the recycling endosome marker TfR (red) (**A**) or the lysosomal marker LAMP1 (red) (**B**). Micrographs were obtained using an SP5 confocal microscope. Graphs show the quantification of co-localization extents using Pearson’s correlation coefficient (n ≥ 10 cells quantified in each of three independent experiments). Unpaired *t*-test, ns *p* > 0.05, * *p* ≤ 0.05, ** *p* ≤ 0.01. For comparison, the colocalization analyses between the wild-type SLC35F1 and F6 proteins with the LAMP1 and TfR markers shown in Figure 2 are replotted in this figure (specified as Figure 2 inside the dedicated columns). The area delimited by the white dotted lines has been enlarged four to five times, and arrows point to SLC35F1-Y367A or SLC35F6-L359A-L360V signals detected at the cell periphery.

As expected, the SLC35F6-L359A-L360V-GFP protein accumulated at the plasma membrane (Figure 6A,B, see arrows) and exhibited a clear diminution of colocalization with the lysosomal marker LAMP1 (Pearson’s coefficient decreased from 0.6402 to 0.4607, as shown by graphs in Figure 6A,B). As previous investigations showed that the Y^333^NGL motif has no effect on mouse SLC35F6 subcellular localization, we decided not to include it in our study [22].

Interestingly, SLC35F1-Y367A-GFP exhibited accumulation at the cell surface, as revealed by microscopic observation (Figure 6A,B, see arrows), and a decreased co-localization level with TfR relative to the wild-type protein (mean Pearson’s correlation coefficients of 0.2631 and 0.6448 for the mutant and wild-type proteins, respectively) (Figure 6A). Colocalization with LAMP1, which was already low, became marginal (0.0989 for the Y367A mutant compared with 0.2758 for its wild-type counterpart) (Figure 6B). By contrast, the second mutant, SLC35F1-Y392A-GFP, was mistargeted to lysosomes, as indicated by its increased co-localization with the lysosomal marker LAMP1 (from 0.2758 for the wild-type protein to 0.6262 for the Y392A mutant) (Figure 6B). It also exhibited decreased co-localization with TfR (from 0.6448 to 0.3831) (Figure 6A).

**Figure 7:**
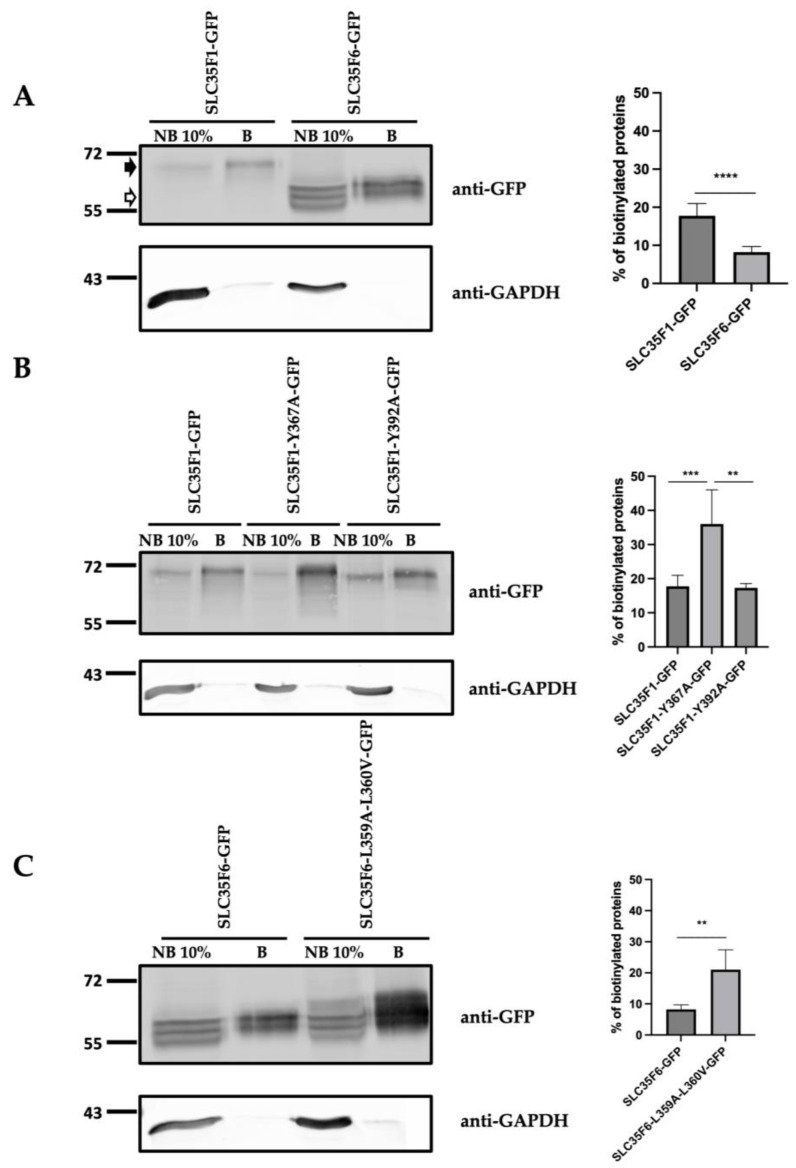
Analysis of the cell surface presence of SLC35F1-GFP and SLC35F6-GFP. (**A**) HeLa cells were transfected with the different constructs for 48 h. Proteins located at the cell surface were then biotinylated and separated from non-biotinylated (i.e., intracellular proteins) using streptavidin agarose beads. The presence of SLC35F1-GFP (~70 kDa, black arrowhead) or SLC35F6-GFP (~60 kDa, white arrowhead) was then analyzed in the B (biotinylated) and NB (non-biotinylated) fractions using western blotting. Of note, 1/10th of the total NB fraction was loaded on the gel. GAPDH detection was used as a negative control. As a cytosolic protein, it should only be detected in the NB fraction. Graphs show the percentages of the protein of interest recovered in the biotinylated fraction (testifying to their membrane localization), relative to the total signal detected in the B and NB fractions (n ≥ 6 independent experiments). Unpaired *t*-test, **** *p* ≤ 0.0001. (**B**) Comparison of the cell surface presence of SLC35F1-GFP and its mutants SLC35F1-Y367A-GFP and SLC35F1-Y392A-GFP, as described in A, with n ≥ 5 independent experiments. Unpaired *t*-test, ** *p* ≤ 0.01, *** *p* ≤ 0.001. (**C**) Comparison of the cell surface presence of SLC35F6-GFP and its mutant SLC35F6-L359A-L360V-GFP, as described in A, except that n ≥ 4 independent experiments were conducted. Unpaired *t*-test, ** *p* ≤ 0.01.

Next, using cell surface biotinylation assays conducted at 4 °C, we determined that wild-type SLC35F1-GFP partly localized to the cell surface under basal conditions (17.75 ± 3.231% of the total SLC35F1-GFP proteins expressed in the cells), whereas less than 10% of the wild-type SLC35F6-GFP population localized at the plasma membrane (8.22 ± 1.46%, *p* < 0.0001 when comparing SLC35F1 and F6) (Figure 7A and Figure S1 showing total expression levels of the proteins of interest 48 h post-transfection). Of note, several bands were detected for human SLC35F6 (which was not the case for mouse SLC35F6 [22]), and the molecular weight of SLC35F6 forms appeared higher in the biotinylated (i.e., plasma membrane) fraction (Figure 7A). One possible explanation is that human SLC35F6 undergoes glycosylation, with the different bands representing forms carrying glycans at different stages of maturation.

Mutation of the Y^367^KQF motif of SLC35F1 increased its plasma membrane presence to 36.04 ± 9.99% (*p* < 0.01), whereas mutation of the Y^392^TSL did not modify its cell surface level (Figure 7). Similarly, mutation of the EQERLL^360^ motif of SLC35F6 resulted in its delocalization to the plasma membrane, as indicated by the increase from 8.22 ± 1.46% to 21.03 ± 6.42% of SLC35F6 proteins found in the biotinylated fraction (*p* < 0.01).

Taken together, our microscopic and biochemical analyses reveal that both tyrosine-based motifs of SLC35F1 and the dileucine motif of SLC35F6 are required for their proper localization to recycling endosomes and lysosomes, respectively. Moreover, we found that mutation of individual tyrosine motifs of SLC35F1 had different consequences on protein distribution, highlighting that these signals control different steps in its intracellular trafficking.

## 3. Discussion

In this study, we first compared the subcellular localization of human SLC35F1 and SLC35F6 proteins and found them to be enriched in different compartments; i.e., Rab11/TfR-positive endosomes and LAMP1-positive late endosomes/lysosomes, respectively. Together with the higher presence of SLC35F1 at the plasma membrane compared with SLC35F6, these results suggest that SLC35F1 may cycle between the cell surface and recycling endosomes, while SLC35F6 mainly travels to lysosomes. Consistent with this interpretation, we found that SLC35F1, but not SLC35F6, accumulates in enlarged Rab11-positive structures upon overexpression of this GTPase.

Next, we identified that the lysosomal sorting of human SLC35F6 is mediated by a conserved cytoplasmic dileucine-based motif (EQERL^359^L^360^), as previously reported for the mouse protein [22]. Since the protein is delocalized to the plasma membrane upon mutation of this motif, and since we have previously shown that expression of a dominant-negative form of Eps15, which disrupts AP-2-mediated endocytosis, does not cause retention of the mouse ortholog at the plasma membrane [22], it is likely that this dileucine motif serves as a sorting signal from the TGN to the endosomal and lysosomal system (via a direct route). After mutation of this EQERL^359^L^360^ motif, SLC35F6 most likely travels to the cell surface by default, as has been reported for several other membrane proteins after mutation of the sorting signals that mediate their direct transport from the TGN to endosomes [31,32]. Interestingly, the intracellular trafficking of SLC35F1 involves two tyrosine-based motifs (Y^367^KQF and Y^392^TSL) located in its C-terminal region. Mutation of the first tyrosine motif (Y367A) increased the presence of SLC35F1 at the plasma membrane and decreased its presence in endosomes, suggesting that this motif either mediates the transport of the protein from the TGN to endosomes by a direct route and/or that it is involved in its internalization from the cell surface when sorted to endosomes by an indirect route. Mutation of the second tyrosine motif (Y392A) of SLC35F1 resulted in its mis-sorting to lysosomes, suggesting that the Y^392^TSL motif plays a role in diverting SLC35F1 from the lysosomal sorting pathway, possibly by favoring its entry into a recycling pathway to the plasma membrane.

It was first proposed that protein recycling to the cell surface is a “default” pathway that occurs owing to bulk membrane flow and thus that it does not involve the recognition of specific recycling signals [33]. For example, it has been reported that the tyrosine-based sorting signals identified in the cytoplasmic region of human TfR (YTRF) and of human Low-Density Lipoprotein (LDL) Receptor (FDNPVY) are involved in the internalization of these proteins but are not required for their recycling to the plasma membrane [34,35]. However, a study later showed that ACAP1 interacts with a phenylalanine-based sequence in the cytoplasmic domain of TfR and that this interaction is required for its recycling to the cell surface [36]. ACAP1 is a member of the Arf6 GTPase-activating protein (GAP) family that orchestrates the formation of recycling tubules from endosomes by regulating Arf6 activity [36,37,38]. Recycling tubules emerge from endosomes upon recruitment of cytosolic membrane curvature sensors, such as the SNX–BAR (Bin–AmphiphysinRvs) complex. Many other cargoes in addition to TfR were then found to be recruited to these tubules, in particular by the retromer complex (with core subunit VPS29, VPS26A/B, and VPS35), as an initial step leading to their recycling to the plasma membrane and/or TGN, including the LDLR [39,40]. Indeed, the initial suggestion that the NPxY motif of this receptor was not involved in its recycling to the cell surface was later challenged by the discovery that this sequence binds to sorting nexin 17 (SNX17), which is part of a retromer-like complex called the “retriever” complex [19,40,41]. Thus, a recycling process may be initiated by specific sorting motifs, the recognition of which may divert the cargo protein from a trafficking route to lysosomes. Our results support that this is the case for the Y^392^TSL motif of SLC35F1, since its mutation induces mis-sorting of the protein to lysosomes.

As a reminder, SLC35F1 and SLC35F6 belong to the Nucleotide Sugar Transporter family. Their subcellular trafficking to endosomes and lysosomes is, therefore, a rather surprising finding, as most members of this family are active in the ER and/or Golgi apparatus. It is worth noting that a large part of lysosomal membrane proteins is heavily glycosylated upon synthesis in the ER and that their glycans form the “lysosomal glycocalyx”, which is thought to protect the membrane of this organelle from the hydrolytic enzymes stored in its lumen [42,43,44]. Interestingly, the presence of glycosyltransferases has been reported in the Mannose 6-Phosphate (M6P) proteome of lysosomes, including GDP-fucose protein O-fucosyltransferase 2, beta 3-glycosyltransferase-like, and sialyltransferase 1 [45,46]. Whether these enzymes are involved in glycocalyx remodeling within lysosomes is currently unknown, but if they are, they would require nucleotide sugars as substrates, possibly delivered via SLC35F1 (which could act in endosomes, from which the nucleotide sugars could be transferred to lysosomes) and/or via SLC35F6 (located in lysosomes). Of note, a metabolomic study of lysosomes reported the presence of nucleotide sugars in the lumen of lysosomes [47], and it was reported that the lysosomal glycocalyx can be trimmed to some extent by luminal glycosydases, such as lysosomal α-mannosidase [48].

Another intriguing idea is that SLC35F1 and/or F6 could promote the storage of nucleotides sugars within endosomes and/or lysosomes and that these could then be released by an exocytic route. It was reported that some glycosyltransferases are active outside of the cells, including ST6Gal-1, which is involved in the sialylation of proteins at the surface of hematopoietic progenitor cells [49]. It was also demonstrated that sialyl-, galactosyl-, and fucosyltransferase activities can remodel glycans at the surface of platelets [50]. The source of extracellular nucleotide sugars for these processes was then identified as nucleotide sugars stored in platelet granules, which can be released in the medium upon platelet activation and subsequent degranulation [11,51,52]. Interestingly, a member of the SLC35 family, SLC35D3, which localizes to platelet-dense granules, was recently found to transport UDP-glucose, supporting a putative role of this transporter in the storage of nucleotide sugars within these granules [8]. It is interesting to consider that SLC35F1 and/or F6 could play similar roles, either in platelets or other cell types, including neurons, since SLC35F1 has been found mainly expressed in the brain.

## 4. Materials and Methods

### 4.1. Materials

Unless otherwise specified, chemicals were obtained from MERCK, Hoeilaart, Belgium. The following primary antibodies were used: goat anti-GFP (Rockland Immunochemicals, Limerick, PA, USA, 600-101-215), mouse anti-LAMP1 (H4A3, DSHB, Iowa City, IA, USA), mouse anti-TfR (13-6800, Invitrogen-Fisher Scientific, Brussels, Belgium), rabbit anti-HA (H6908), mouse anti-GAPDH (G8795) and rabbit anti-SLC35F6 (HPA034655) (Sigma-Aldrich, Hoeilaart, Belgium), and rabbit anti-SLC35F1 (Origene, Herford, Germany, TA345756). IRDye^®^ secondary antibodies 680 or 800 for western blotting and Alexa Fluor^TM^-coupled secondary antibodies 488 or 568 for immunofluorescence were obtained from LI-COR Biosciences (Lincoln, NE, USA), and Invitrogen-Fisher Scientific (Brussels, Belgium), respectively. Cell culture media, trypsin, and antibiotics were obtained from BIOWEST (Nuaillé, 49340 France) and fetal bovine serum from MERCK (Hoeilaart, Belgium).

### 4.2. DNA Constructs

The human SLC35F1-HA plasmid was obtained from Origene (Herford, Germany) (SLC35F1_OHu24322C_pcDNA3.1(+)-C-HA, NM_001029858.4).

The coding sequence of human SLC35F6 (Q8N357) was synthesized by Genscript (Piscataway, NJ, USA) and inserted in a pET-14b plasmid using the Express cloning service.

The sequence coding for human SLC35F1 or SLC35F6 was amplified using InFusion primers (according to the InFusion^TM^ HD Cloning methods, Takara Bio Inc., Saint-Germain-en-Laye, France) and then inserted in a pEGFP_N3 plasmid via BamH1 (for *SLC35F6*) or BglII (for *SLC35F1*) restriction sites. The SLC35F1-Y367A mutant was generated by site-directed mutagenesis using PCR. The SLC35F1-Y392A, SLC35F1-F6c (SLC35F1 with C-terminal tail of SLC35F6), SLC35F6-F1c (SLC35F6 with C-terminal tail of SLC35F1), and SLC35F6-L359A-L360V mutant coding sequences were obtained by gBlocks^TM^ production (Integrated DNA Technologies, Leuven, Belgium) and then inserted in a pEGFP_N3 plasmid via BamH1 restriction site using InFusion^TM^ HD Cloning methods.

The human DsRed-Rab11 WT was a gift from Richard Pagano (Addgene plasmid #12679; http://n2t.net/addgene:12679; RRID:Addgene_12679 [53]) (accessed on 20 May 2024).

### 4.3. Cell Culture and Transient Transfection

HeLa cells (CCL-2 from ATCC, American Type Culture Collection, Manassas, VA, USA) were cultured in DMEM medium supplemented with 10% fetal bovine serum and 1% of a 100× Penicillin–Streptomycin solution (6.03 g/L Penicillin G Sodium Salt, 10 g/L Streptomycin Sulfate) at 37 °C in a humidified atmosphere with 5% CO_2_. SH-SY5Y (ATCC CRL-2266) were cultured in DMEM-F12 supplemented with 10% of heat-inactivated fetal bovine serum and with 1% of a 100× Penicillin–Streptomycin solution. Transient transfections of plasmids were conducted using FuGENE^®^ HD reagent (Promega, Madison, WI, USA) following the manufacturer’s instructions.

### 4.4. Western Blotting

Cell lysates prepared in a RIPA buffer [50 mM Tris/HCl (pH 7.4), 120 mM NaCl, 1% (*v*/*v*) Triton X-100, 0.1% SDS, and 1% deoxycholate] containing protease inhibitors (Complete Mini Protease inhibitor cocktail tablets, Roche, commercialized by MERCK, Hoeilaart, Belgium) were mixed with Laemmli’s sample buffer containing di-thiothreitol (DTT, final concentration of 100 mM) and resolved using Sodium Dodecyl Sulfate-PolyAcrylamide Gel Electrophoresis (SDS-PAGE) next to a Color Prestained Protein Standard (Broad Range) from NEW ENGLAND BioLabs (Ipswich, MA, USA). After transfer on a low fluorescence polyvinylidene fluoride membrane (Immobilon-FL, Sigma-Aldrich, Hoeilaart, Belgium) and blocking in 10% fat-free milk in PBS-Tween 0.1%, proteins of interest were detected using specific primary antibodies (see Section 4.1) diluted (1000 times for anti-GFP and 2000 times for anti-GAPDH) in PBS-Tween 0.1% containing 5% fat-free milk, followed by incubation with corresponding IRDye^®^ secondary antibodies (diluted 10,000 times). Infrared signals were detected by an Amersham^TM^ Typhoon^TM^ Biomolecular Imager (Buckinghamshire, UK) and quantified using the Image Studio™ Lite Quantification Software, version 5.2.5 (the sum of all bands detected for SLC35F6 was used in quantifications).

### 4.5. Immunofluorescence

At 48 h post-transfection, HeLa or SH-SY5Y cells cultured on 15-mm round glass coverslips were fixed with 4% paraformaldehyde in PBS for 10 min at room temperature and then incubated with 0.5% saponin/PBS for 10 min at room temperature. After blocking with 1% BSA in PBS, the cells were incubated for 1 h with primary antibodies (diluted 100 times) and for 1 h with Alexa Fluor^TM^-conjugated secondary antibodies (diluted 250 times) (see Section 4.1 for antibody references). The coverslips were mounted using Mowiol. Confocal images were taken with a Leica SP5 microscope (Leica Microsystems, Wetzlar, Germany), using a 40× or 60× objective and 488 and 561 lasers, combined with 500–535 and 567–618 emission ranges, respectively. At least 10 images were taken in each of the experiments, which were conducted in triplicate. Pearson’s correlation coefficients were measured using the Leica software (Leica Application Suite 2.7.3.9723). Means ± SD were calculated and the statistical relevance of differences between conditions was assessed using an unpaired *t*-test.

### 4.6. Cell Surface Biotinylation Assay

Transfected HeLa cells were washed several times over a 30-min period with ice-cold PBS supplemented with 0.7 mM CaCl_2_ and 0.25 mM MgSO_4_ (PBS++, pH 8). Cell-surface proteins were then labeled by incubation of the cells with 1 mg/mL sulfo-NHS-SS-biotin [sulfosuccinimidyl 2-(biotinamido)ethyl-1,3-dithiopropionate] (Pierce, commercialized by Invitrogen-Fisher Scientific, Brussels, Belgium) in PBS++ for 50 min on ice. Biotinylation was stopped by several washes with ice-cold 100 mM NH_4_Cl/PBS++, and the cells were lysed with RIPA buffer. Next, biotinylated proteins were precipitated with streptavidin–agarose beads (Pierce, Invitrogen-Fisher Scientific, Brussels, Belgium) by centrifugation at 4 °C for 1 min 30 at 8300× *g* in a benchtop centrifuge. The non-biotinylated proteins were recovered (i.e., the supernatant was collected). After several washes of the beads with ice-cold PBS++ using centrifugation in the same conditions mentioned above, biotinylated proteins were eluted by incubation for 5 min at 70 °C in 2× Laemmli’s buffer containing 200 mM fresh DTT. One-tenth of the supernatant containing non-biotinylated proteins, and proteins eluted from the beads (biotinylated), were separated using SDS-PAGE (10% gel), and the proteins of interest were detected by western blotting as described above.

## Data Availability

All data used in this article are included within the figures and Supplementary Materials.

## Supplementary Materials

The following supporting information can be downloaded at: https://www.mdpi.com/article/10.3390/ijms25126718/s1.
