# Supplementary material for: Residence of the Nucleotide Sugar Transporter Family Members SLC35F1 and SLC35F6 in the Endosomal/Lysosomal Pathway
Source: Int J Mol Sci. 2024 Jun 18;25(12):6718. doi: 10.3390/ijms25126718 (PMC11203873; doi:10.3390/ijms25126718)
Supplement: Supplementary file 1 [file ijms-25-06718-s001.zip › ijms-3053807-supplementary.pdf]

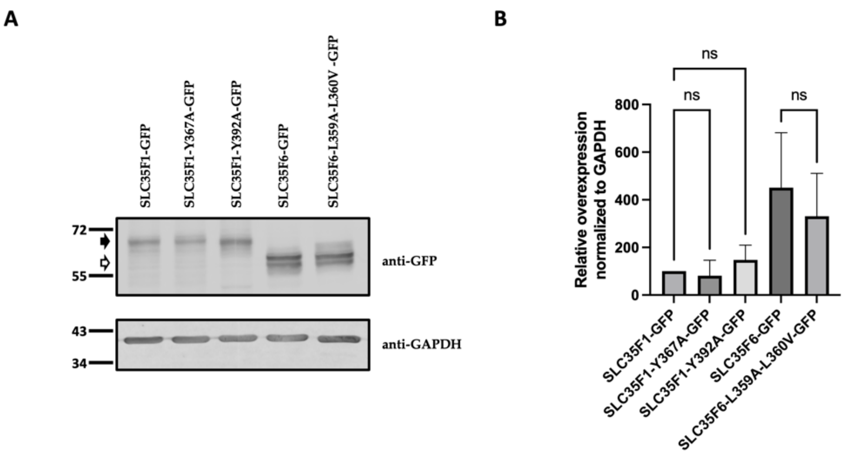

Figure S1.

(A) Analysis of expression of the different constructs by western blotting after transfection in HeLa cells. Proteins were extracted 48h post-transfection and resolved by SDS-PAGE. An anti-GFP antibody was used to detect the tagged proteins SLC35F1-GFP, SLC35F1-Y367A-GFP, SLC35F1-Y392A-GFP (~70kDa, black arrowhead), as well as SLC35F6-GFP and SLC35F6-L359A-L360V-GFP (~60kDa, white arrowhead). GAPDH detection was used as a loading control. **(B) Quantifications of the relative expression of the mutants relative to their wild-type counterparts.** n = 4 independent experiments were quantified, unpaired t-test, ns $p > 0.05$.
